# Supplementary material for: Surgical capacity, productivity and efficiency at the district level in Sub-Saharan Africa: A three-country study
Source: PLoS One. 2022 Nov 30;17(11):e0278212. doi: 10.1371/journal.pone.0278212 (PMC9710758; doi:10.1371/journal.pone.0278212)
Supplement: S1 Table — (PDF) [file pone.0278212.s001.pdf]

S2 Table. Full result of DEA by country

Country	Hospital code	Efficiency	Tech. eff.	Pure tech. eff.	Scale eff.	RTS
Tanzania	103	1.00	1.00	1.00	1.00	CRS
Tanzania	104	0.62	0.62	1.00	1.00	CRS
Tanzania	105	0.96	0.98	1.00	0.97	IRS
Tanzania	106	1.00	1.00	1.00	1.00	CRS
Tanzania	107	0.66	0.70	1.00	0.94	IRS
Tanzania	108	0.46	0.46	1.00	1.00	CRS
Tanzania	109	0.84	0.85	1.00	1.00	IRS
Tanzania	110	0.99	1.00	1.00	0.99	IRS
Tanzania	111	0.79	1.00	1.00	0.79	IRS
Tanzania	114	0.63	0.63	1.00	1.00	IRS
Tanzania	119	1.00	1.00	1.00	1.00	CRS
Tanzania	120	1.00	1.00	1.00	1.00	CRS
Tanzania	124	1.00	1.00	1.00	1.00	CRS
Tanzania	125	0.59	0.60	1.00	0.99	IRS
Tanzania	127	1.00	1.00	1.00	1.00	CRS
Tanzania	130	0.29	0.29	0.32	1.00	IRS
Tanzania	135	0.82	1.00	1.00	0.82	IRS
Tanzania	136	0.40	0.43	1.00	0.94	IRS
Malawi	201	0.80	0.81	0.91	0.99	IRS
Malawi	202	0.12	0.12	0.14	1.00	CRS
Malawi	203	0.85	1.00	1.00	0.85	IRS
Malawi	204	0.33	0.33	1.00	1.00	CRS
Malawi	205	0.83	0.83	1.00	1.00	IRS
Malawi	206	0.88	1.00	1.00	0.88	IRS
Malawi	207	0.89	0.90	1.00	0.99	IRS
Malawi	208	0.80	0.80	1.00	1.00	CRS
Malawi	209	0.11	0.11	1.00	1.00	CRS

Malawi	210	1.00	1.00	1.00	1.00	CRS
Malawi	211	1.00	1.00	1.00	1.00	CRS
Malawi	212	1.00	1.00	1.00	1.00	CRS
Malawi	213	0.43	0.43	1.00	1.00	CRS
Malawi	214	0.72	0.72	1.00	1.00	IRS
Malawi	215	0.35	0.35	1.00	0.98	IRS
Malawi	216	0.57	0.62	1.00	0.92	IRS
Malawi	217	0.19	0.19	1.00	1.00	CRS
Malawi	218	0.97	1.00	1.00	0.97	IRS
Malawi	219	0.65	0.65	1.00	1.00	CRS
Malawi	220	0.81	0.81	1.00	1.00	CRS
Malawi	221	1.00	1.00	1.00	1.00	CRS
Malawi	222	1.00	1.00	1.00	1.00	CRS
Zambia	301	0.11	0.12	1.00	0.98	IRS
Zambia	302	0.38	0.39	1.00	0.96	IRS
Zambia	304	1.00	1.00	1.00	1.00	CRS
Zambia	305	0.30	0.30	1.00	1.00	CRS
Zambia	306	0.04	0.04	0.05	1.00	CRS
Zambia	307	0.44	0.44	1.00	1.00	CRS
Zambia	309	0.62	0.62	1.00	1.00	CRS
Zambia	310	1.00	1.00	1.00	1.00	CRS
Zambia	312	0.41	0.41	1.00	1.00	CRS
Zambia	314	0.36	0.36	1.00	0.99	IRS
Zambia	315	0.55	0.55	1.00	1.00	CRS
Zambia	317	0.61	0.62	1.00	0.98	IRS
Zambia	318	0.10	0.10	1.00	0.99	IRS
Zambia	319	0.15	0.15	0.20	1.00	CRS
Zambia	320	0.17	0.17	0.19	0.99	IRS
Zambia	321	0.60	0.60	1.00	1.00	CRS

Zambia	322	0.37	0.37	0.55	1.00	IRS
Zambia	323	0.96	1.00	1.00	0.96	IRS
Zambia	325	0.17	0.17	0.25	1.00	CRS
Zambia	326	0.25	0.25	0.35	1.00	CRS
Zambia	327	0.38	0.43	1.00	0.86	IRS

Note: RTS refers to returns to scale. IRS – increasing returns to scale (marginal productivity equals to 1). CRS – constant returns to scale (marginal productivity equals to 0).
