# Supplementary material for: Surgical capacity, productivity and efficiency at the district level in Sub-Saharan Africa: A three-country study
Source: PLoS One. 2022 Nov 30;17(11):e0278212. doi: 10.1371/journal.pone.0278212 (PMC9710758; doi:10.1371/journal.pone.0278212)
Supplement: S2 Table — (PDF) [file pone.0278212.s002.pdf]

S3 Table. Average value of input slacks by efficiency and country

Country	Efficiency	Personnel	Infrastructure	Procedure	Equipment	Supplies
**Tanzania**	**e<1**	0.795	1.047	3.146	0.724	3.613
	**e<0.5**		0.505	2.909	0.594	1.438
**Malawi**	**e<1**	1.337	1.167	2.160	2.281	3.344
	**e<0.5**	0.727	0.585	0.839	1.378	0.931
**Zambia**	**e<1**	0.640	0.716	2.722	1.676	1.866
	**e<0.5**	0.640	0.613	2.143	1.355	1.420

Note: Hospitals were divided into two categories based on the DEA efficiency score: low efficiency (e<1) and extremely low efficiency (e<0.5).
