# Supplementary material for: Surgical capacity, productivity and efficiency at the district level in Sub-Saharan Africa: A three-country study
Source: PLoS One. 2022 Nov 30;17(11):e0278212. doi: 10.1371/journal.pone.0278212 (PMC9710758; doi:10.1371/journal.pone.0278212)
Supplement: S3 Table — (PDF) [file pone.0278212.s003.pdf]

S4 Table. Relationship between surgical productivity and production factors – quantile regression at 25th, 50th, and 75th percentiles

Variables	25th - DLH	25th - Country	50th - DLH	50th - Country	75th - DLH	75th - Country
Personnel	8.547***	5.916***	10.145***	5.632***	13.017***	5.068**
	(1.656)	(2.118)	(1.653)	(1.931)	(1.588)	(2.018)
Infrastructure	1.276	3.450	-9.931	1.158	-2.271	2.900
	(7.455)	(8.493)	(7.442)	(8.516)	(7.151)	(7.346)
Procedures	-0.278	-1.203	1.378	-0.084	0.796	1.907
	(2.911)	(1.386)	(2.906)	(2.131)	(2.792)	(2.247)
Equipment	-0.581	-1.303	-1.356	1.882	5.294	4.154
	(4.198)	(3.104)	(4.191)	(2.570)	(4.027)	(3.014)
Supplies	1.270	0.441	2.929	1.285	1.308	-1.125
	(2.443)	(1.068)	(2.439)	(1.417)	(2.343)	(1.607)
Tanzania		Ref		Ref		Ref
Malawi		32.461		83.771*		93.377**
		(38.861)		(45.495)		(41.312)
Zambia		-6.483		-30.140		-57.559**
		(21.515)		(25.855)		(22.823)
Constant	-14.688	28.958	51.629	-1.580	-76.500	-13.430
	(108.236)	(62.007)	(108.053)	(70.526)	(103.821)	(86.679)
Observations	61	61	61	61	61	61
R-squared	0.235	0.259	0.346	0.393	0.42	0.492

Notes: Standard errors clustered at hospital/country level shown in parentheses. *** p<0·01, ** p<0·05, * p<0·1.
