# Supplementary material for: Surgical capacity, productivity and efficiency at the district level in Sub-Saharan Africa: A three-country study
Source: PLoS One. 2022 Nov 30;17(11):e0278212. doi: 10.1371/journal.pone.0278212 (PMC9710758; doi:10.1371/journal.pone.0278212)
Supplement: S4 Table — (PDF) [file pone.0278212.s004.pdf]

S5 Table. Relationship between surgical efficiency and production factors using Tobit and two-stage bootstrap models

Variables	Tobit-DLH	Tobit-Country	Variables	BS-DLH	BS-Country
Personnel	0.017**	0.013***	Personnel	0.021***	0.008
	(0.007)	(0.002)		(0.005)	(0.006)
Infrastructure	0.016	0.006	Infrastructure	0.002	0.015
	(0.031)	(0.014)		(0.018)	(0.019)
Procedures	-0.013	-0.012	Procedures	-0.011	-0.016**
	(0.010)	(0.009)		(0.007)	(0.007)
Equipment	-0.019	-0.008	Equipment	-0.017	-0.019*
	(0.017)	(0.026)		(0.011)	(0.010)
Supplies	-0.010	-0.009	Supplies	0.004	0.009
	(0.010)	(0.005)		(0.006)	(0.006)
Tanzania		Ref	Tanzania		Ref
Malawi		-0.181***	Malawi		0.175*
		(0.050)			(0.093)
Zambia		-0.297***	Zambia		-0.174**
		(0.072)			(0.081)
Constant		1.241***	Constant	0.628**	0.726***
		(0.335)		(0.270)	(0.267)
Observations	61	61	Observations	61	61
R-squared	0.191	0.283	P-value	0.000	0.000

Notes: Standard errors clustered at hospital/country level shown in parentheses. BS – two-stage bootstrap model. *** p<0·01, ** p<0·05, * p<0·1.
