# Supplementary material for: Surgical capacity, productivity and efficiency at the district level in Sub-Saharan Africa: A three-country study
Source: PLoS One. 2022 Nov 30;17(11):e0278212. doi: 10.1371/journal.pone.0278212 (PMC9710758; doi:10.1371/journal.pone.0278212)
Supplement: S1 File — (PDF) [file pone.0278212.s005.pdf]

S1. Data Envelopment Analysis

Data Envelopment Analysis

A model proposed by Chames, Cooper and Rhodes (CCR) [1] is used for generating the efficiency score. Assume there are n district level hospitals (DLHs) producing s outputs, $y_{rj}, r = 1, 2, \dots s; j = 1, 2, \dots n$, with m inputs, $x_{ij}, i = 1, 2, \dots m; j = 1, 2, \dots n$, which are set up as follows

$$X_j = \left(x_{1j}, x_{2j,}, \dots, x_{mj}\right)^T$$

$$Y_j = \left(y_{1j}, y_{2j,}, \dots, y_{sj}\right)^T$$

$$v = (v_1, v_{2,}, \dots, v_m)^T$$

$$u = (u_1, u_2, \dots, u_s)^T$$

The efficiency score is expressed as

$$\theta_j = \frac{u^T Y_j}{v^T X_j}, j = 1, 2, \dots, n \tag{1}$$

Both X_j and Y_j are given and the target is to select input and output weights, v and u to achieve $\theta_j \leq 1$. For each DLH, the fractional programming problem of an output-oriented model is as follows

$$min \ \frac{v^T X_j}{u^T Y_j} = \frac{\sum_{i=1}^{m} v_i x_{ij}}{\sum_{r=1}^{s} u_r y_{rj}} \tag{2}$$

$$s.t. \ \frac{\sum_{i=1}^{m} v_i x_{ij}}{\sum_{r=1}^{s} u_r y_{rj}} \geq 1, j = 1, 2, \dots, n$$

$$u_r \geq 0, \ v_i \geq 0$$

For DH_{j0}, we assume that $t = \frac{1}{v^T X_0}$, $\omega = tv$ and $\mu = tu$, the Equation (3) can be expressed in the following linear programming problem

$$min \ \omega^T X_0 \tag{3}$$

$$s.t. \ \omega^T X_j - \mu^T Y_j \geq 0, j = 1, 2, \dots, n$$

$$\mu^T Y_0 = 1$$

$$\omega \geq 0, \mu \geq 0$$

A two-stage DEA is used to identify all efficiency slacks:

$$\max \theta + \varepsilon \sum (s^+ + s^-) \tag{4}$$

$$s.t. \sum_{j=1}^{n} \lambda_j x_{ij} + s_i^- = x_{ik}$$

$$\sum_{j=1}^{n} \lambda_j y_{rj} - s_r^+ = \theta y_{rk}$$

$$\sum_{j=1}^{n} \lambda_j = 1$$

$$\lambda \geq 0;\ s^- \geq 0;\ s^+ \geq 0$$

$$i = 1,2,\dots,m;\ j = 1,2,\dots,n;\ r = 1,2,\dots,s$$

in which λ is the coefficient of the linear combination and ε is a non-Archimedean element, $\varepsilon > 0$. A district hospital is DEA efficient if $\theta^* = 1$ and $s^{+*} = s^{-*} = 0$ for all i and r. Here, the slack means the excessive use of inputs should be reduced without sacrificing current efficiency. For hospital j the value of the slack, s_i^-, indicates the amount of the input i can be reduced, in order to maintain the current output level but at a more efficient level, which can be viewed as the reason for inefficiency and the extent to which improvement should be achieved.

Efficiency scores under the variable return to scale [2] are also calculated, from which the pure technical efficiency, scale efficiency and the returns to scale can be assessed. A hospital is DEA efficient when all efficiency scores are equal to one, otherwise it is weakly DEA efficient. The type of returns to scale explains the variation between input and output variables, i.e. whether the increase in input can lead to increasing (IRS), constant (CRS), or decreasing (DRS) change in production.

However, one potential weakness of the CCR model is the zero-weighted inputs that the DEA model chooses the most relevant input but ignores the least relevant one. All five inputs (production factors) – personnel, infrastructure, procedure, equipment and supplies – are necessary to an operation. Hence, the common-set of weights (CSW) method was selected to calculate the optimal input weights, which was defined by S. Saati in 2008 [3]. Rather than assigning different weights for different DLHs, the CSW-DEA set the same weights for all DLHs until at least one DLH reaches the optimal production level. Firstly the restriction on weights for all DLHs is set as lower and upper bonds of inputs and outputs. The upper bounds of input and output weights are the reciprocals of the maximum value of each input/output of all DLHs. The lower bounds of all weights is set to zero. Under the assumption of the same deviation between upper and lower bounds, the bounds of input and output weights can be

expressed as a proportion of the weight intervals of each input and output. Then the optimal weights are the solution when at least one DLH reaches the efficient level (efficiency=1).

Estimation of hospital efficiency

One weakness of the DEA is the lack of strong tests for model specification [4]. To mitigate that, two follow-up tests were included for estimating the relationship between hospital efficiency and input variables. The first was a Tobit regression using externally generated efficiency scores. The DEA score, generated from the Stata DEA package by Lee and Ji [5], was calculated by country. Since the efficiency score ranges from zero to one, the model was set as left-censoring at zero and right-censoring at one, using five inputs. Two models with and without country dummy variables were included by setting Tanzania again as the reference country. Standard errors were clustered at the hospital and country level.

The second follow-up test, a two-stage bootstrap regression by Simar and Wilson [6], was performed to identify the determinants of hospital efficiency, which provided more accurate estimates by correcting the finite sample bias and inconsistency in the estimation process. There were two loops in calculating bias: corrected efficiency scores and bootstrap estimates of parameters and coefficients. Because of the small sample size, efficiency scores were generated for the full sample rather than by country.

Reference

1. Charnes A, Cooper WW, Rhodes E. Measuring the efficiency of decision making units. European journal of operational research. 1978;2(6):429-44.

2. Banker RD, Thrall RM. Estimation of returns to scale using data envelopment analysis. European Journal of operational research. 1992;62(1):74-84.

3. Saati S. Determining a common set of weights in DEA by solving a linear programming. Journal of Industrial Engineering, International. 2008;4(6):51-6.

4. Cubbin J, Tzanidakis G. Regression versus data envelopment analysis for efficiency measurement: an application to the England and Wales regulated water industry. Utilities policy. 1998;7(2):75-85.

5. Lee C, Ji Y-b, editors. Data envelopment analysis in Stata. Stata Conference DC; 2009: Citeseer.

6. Simar L, Wilson PW. Estimation and inference in two-stage, semi-parametric models of production processes. Journal of econometrics. 2007;136(1):31-64.
