# Supplementary material for: Surgical capacity, productivity and efficiency at the district level in Sub-Saharan Africa: A three-country study
Source: PLoS One. 2022 Nov 30;17(11):e0278212. doi: 10.1371/journal.pone.0278212 (PMC9710758; doi:10.1371/journal.pone.0278212)
Supplement: Attachment — Submitted filename: Report PONE-D-22-07365.pdf [file pone.0278212.s007.pdf]

Title: Surgical capacity, productivity and efficiency at the district level in Sub-Saharan Africa: a three-country study

Manuscript Number: PONE-D-22-07365
Journal: PLOS ONE

This paper deals with the determinants of hospital efficiency in district hospitals in three African countries, Tanzania, Malawi, and Zambia.

The authors determine the hospital efficiency scores for three counties. They discussed that hospital efficiency, combining capacity measures and surgical outputs, is a better indicator of surgical performance than capacity measures, which could be misleading if used alone for surgical planning. They concluded that investment in the surgical workforce, in particular, plays a central role in improving district hospital surgical productivity and efficiency.

Overall, the aim of this paper may be attractive for the practitioner of the DEA model; but first, the authors should address the following comments.

1. One of the features of the DEA models is that of providing the benchmark (target unit) for inefficient units, While the authors do not discuss the key concept of target unit for inefficient DMUs. The authors must suggest the target unit for inefficient DMUs to learn and improve by following it.
2. **P. 6 L. 1 Hospital efficiency**
3. The authors obtain the efficiency scores using the constant returns to scale (CRS) DEA model while utilizing the variable returns to scale (VRS) DEA model would be more common.
4. **P. 6 L. 7 Typo**

Chames et al. → Charnes et al.

5. **P. 12 L. 2 Congestion in the resources**
   The authors state as follows:

> Our findings question this assumption, demonstrating that surgical production factors do not contribute equally to outputs; and there can even be an inverse relationship between surgical capacity and efficiency. All hospitals that scored high on surgical capacity (PIPES index) scored low for efficiency, which suggests that the provision of more resources to a hospital may not make it more productive.

The above statements show that the congestion may be present in the inputs. The authors must deal with identifying and managing congestion using DEA.

6. **P. 14 L. 6 The mortality rate**

The authors state as follows:
“The mortality rate in the sample was very low (0.08%), hence it is excluded in the DEA.” The mortality rate in the concept of DEA is an undesirable output that should be considered in the efficiency score of the hospitals.

7. **S1 Table. Full result of DEA by country**
   The authors only identify the returns to scale (RTS) status of hospitals without any interpretation. Why do all DMUs show constant RTS or increasing RTS? The authors must discuss this concept in detail.
