# Supplementary material for: Surgical capacity, productivity and efficiency at the district level in Sub-Saharan Africa: A three-country study
Source: PLoS One. 2022 Nov 30;17(11):e0278212. doi: 10.1371/journal.pone.0278212 (PMC9710758; doi:10.1371/journal.pone.0278212)
Supplement: Attachment — Submitted filename: Response to Reviewers.docx [file pone.0278212.s008.docx]

**Editors’ comments**

1. Please ensure that your manuscript meets PLOS ONE's style requirements, including those for file naming.

Thank you for your comments. The manuscript and files are updated.

2. Please include a complete copy of PLOS’ questionnaire.

Thank you for your comments. Please check the questionnaire uploaded with the revised manuscript.

3. In ethics statement in the manuscript and in the online submission form, please provide additional information about the patient records/samples used in your retrospective study. Specifically, please ensure that you have discussed whether all data/samples were fully anonymized before you accessed them and/or whether the IRB or ethics committee waived the requirement for informed consent. If patients provided informed written consent to have data/samples from their medical records used in research, please include this information.

The ethnics statement is updated in the manuscript.

“Prior Ministry of Health approval for data collection and informed audio-recorded consent for interviews from respondents were obtained. All of the approving research ethics committees (RECs) waived the requirement for writ-ten informed consent. Ethical approval was granted by the REC of the Royal College of Surgeons in Ireland, the project consortium lead, under approval No. REC 1417. In the implementation countries, ethical approval was received from the College of Medicine Research Ethics Committee in Malawi (approval No. P.05/17/2179), the University of Zambia Biomedical Research Ethics Committee (approval No. 005-05-17), the Kilimanjaro Christian Medical College Research Ethics and Review Committee (approval No. CRERC 2026), and the National Institute for Medical Research in Tanzania (approval No. NIMR/HQ/R.8a/Vol. IX/2600).”

4. In your Data Availability statement, you have not specified where the minimal data set underlying the results described in your manuscript can be found. PLOS defines a study's minimal data set as the underlying data used to reach the conclusions drawn in the manuscript and any additional data required to replicate the reported study findings in their entirety. All PLOS journals require that the minimal data set be made fully available. For more information about our data policy, please see <http://journals.plos.org/plosone/s/data-availability>.

Upon re-submitting your revised manuscript, please upload your study’s minimal underlying data set as either Supporting Information files or to a stable, public repository and include the relevant URLs, DOIs, or accession numbers within your revised cover letter. For a list of acceptable repositories, please see <http://journals.plos.org/plosone/s/data-availability#loc-recommended-repositories>.

Any potentially identifying patient information must be fully anonymized.

Important: If there are ethical or legal restrictions to sharing your data publicly, please explain these restrictions in detail. Please see our guidelines for more information on what we consider unacceptable restrictions to publicly sharing data: <http://journals.plos.org/plosone/s/data-availability#loc-unacceptable-data-access-restrictions>. Note that it is not acceptable for the authors to be the sole named individuals responsible for ensuring data access.

We will update your Data Availability statement to reflect the information you provide in your cover letter.

Thank you for your comments. The minimal dataset is prepared and uploaded with the revised manuscript (Supporting information-Compressed file).

5. Thank you for stating the following financial disclosure:

“The project is funded by the European Union’s Horizon 2020 research and innovation programme under the grant agreement No. 733391. Salaries of JG, MZ, and CP were paid by this grant. Cost of the study was covered by this grant.”

Please state what role the funders took in the study. If the funders had no role, please state: "The funders had no role in study design, data collection and analysis, decision to publish, or preparation of the manuscript."

If this statement is not correct you must amend it as needed.

Please include this amended Role of Funder statement in your cover letter; we will change the online submission form on your behalf.

Thank you for your comments. The statement and the cover letter are updated. Please check the new files.

6. Please include captions for your Supporting Information files at the end of your manuscript, and update any in-text citations to match accordingly. Please see our Supporting Information guidelines for more information: <http://journals.plos.org/plosone/s/supporting-information>

Thank you for your comments. Please check the new manuscript.

7. We note that Figure 2 in your submission contain map images which may be copyrighted. All PLOS content is published under the Creative Commons Attribution License (CC BY 4.0), which means that the manuscript, images, and Supporting Information files will be freely available online, and any third party is permitted to access, download, copy, distribute, and use these materials in any way, even commercially, with proper attribution. For these reasons, we cannot publish previously copyrighted maps or satellite images created using proprietary data, such as Google software (Google Maps, Street View, and Earth). For more information, see our copyright guidelines: http://journals.plos.org/plosone/s/licenses- and-copyright.

We require you to either (1) present written permission from the copyright holder to publish these figures specifically under the CC BY 4.0 license, or (2) remove the figures from your submission.

Thank you for your comments. Please check the signed permission forms uploaded separately (labelled as Other).

**Comments from reviewers**

**Reviewer 1**

*This paper deals with the determinants of hospital efficiency in district hospitals in three African countries, Tanzania, Malawi, and Zambia.*

*The authors determine the hospital efficiency scores for three counties. They discussed that hospital efficiency, combining capacity measures and surgical outputs, is a better indicator of surgical performance than capacity measures, which could be misleading if used alone for surgical planning. They concluded that investment in the surgical workforce, in particular, plays a central role in improving district hospital surgical productivity and efficiency.*

*Overall, the aim of this paper may be attractive for the practitioner of the DEA model; but first, the authors should address the following comments:*

*One of the features of the DEA models is that of providing the benchmark (target unit) for inefficient units, While the authors do not discuss the key concept of target unit for inefficient DMUs. The authors must suggest the target unit for inefficient DMUs to learn and improve by following it.*

Thank you for the comments. There is no reference point for inefficient facilities, hence our approach needed to be adjusted. Rather than focusing on specific inefficient hospitals, we highlight the problems of hospitals with low efficiency (DEA score < 0.5). As what’s shown in Table 4, compared with highly efficient hospitals, those hospitals had lower values scores in surgical staff but relatively higher score in supplies, which were consistent in three countries. Hence, in Discussion we suggested that investment in surgical staff would be a better choice for the national surgical planning.

*P. 6 L. 1 Hospital efficiency*

*The authors obtain the efficiency scores using the constant returns to scale (CRS) DEA model while utilizing the variable returns to scale (VRS) DEA model would be more common.*

Thank you for your comments. Yes, the use of VRS can provide additional details. Due to the text length requirement, we haven’t included the VRS results in the main context but in the Supporting Information where we also presented the details of efficiency score using variable returns to scale and the nonincreasing returns to scale.

P. 6 L. 7 Typo

Chames et al. → Charnes et al.

Thank you for pointing it out. It has been corrected.

*P. 12 L. 2 Congestion in the resources*

*The authors state as follows:*

*“Our findings question this assumption, demonstrating that surgical production factors do not contribute equally to outputs; and there can even be an inverse relationship between surgical capacity and efficiency. All hospitals that scored high on surgical capacity (PIPES index) scored low for efficiency, which suggests that the provision of more resources to a hospital may not make it more productive.”*

*The above statements show that the congestion may be present in the inputs. The authors must deal with identifying and managing congestion using DEA.*

Thank you for your comments. The DEA efficiency suggests the best allocation of different inputs under certain assumptions given the current output level. Slacks that indicate the over input will lead to inefficiency. Due to the length requirement, we haven’t included the slacks discussion in the paper, because the core interest of this paper is to show the difference in measuring hospital performance (the difference in input weights using different measurement), thereby drawing more attention to the measurement selection and healthcare development at the district level. In addition, the common set of weights (CSW) in DEA was included to improve the accuracy of the input weights, the result of which was shown in Table 5. The result of input slacks are added into S3 Data envelopment analysis result in Supporting Information.

*P. 14 L. 6 The mortality rate*

*The authors state as follows:*

*“The mortality rate in the sample was very low (0.08%), hence it is excluded in the DEA.” The mortality rate in the concept of DEA is an undesirable output that should be considered in the efficiency score of the hospitals.*

We would like to point out that the low mortality rate is very low in the sample of hospitals in our study, and it is rather homogeneous across the sample. If 99.2% of DMUs have no morality rate, then there is little difference in the output (mortality) and we cannot run the DEA regression for a reliable result.

*S1 Table. Full result of DEA by country*

*The authors only identify the returns to scale (RTS) status of hospitals without any interpretation. Why do all DMUs show constant RTS or increasing RTS? The authors must discuss this concept in detail.*

Thank you for your comments. We have added an explanation for the DEA results. Table S1 shows the efficiency score under the constant returns to scale, variable returns to scale, and the nonincreasing returns to scale. The Stata command also calculates the returns to scale (the last column of Table S1) by calculating the marginal productivity. If the marginal productivity equals to 1, it is IRS. If the marginal productivity equals to 0, it is CRS. All related outputs were shown in Table S1. The explanation about the marginal values has been added to the table note.

**Reviewer 2**

*This paper presents the application of data envelopment analysis (DEA) in the hospital ward. In fact, this paper investigates the determinants of hospital efficiency in district hospitals in three African countries. For this purpose, the initial DEA models (CCR, BCC) have been used.*

*Here are some of my comments on the paper:*

*1) The DEA literature needs to be better articulated. References need to be updated.*

*2) Explain the reasons for choosing production indicators. Input factors must also be stated.*

*3) For better results, I suggest using the SBM-model as well*

Thank you for your comments. We have addressed them in the new version of the manuscript.

1) Except the reference of the methodology and command, other DEA related references are replaced by the new publications.
2) The production factors are the factors used in the PIPES index survey defined by an organisation name Surgeons Overseas. In order to compare the difference in using different measures for measuring production performance, these five production factors were included in the DEA regression. The output factor used in the DEA is the surgical volume, which is also widely used as an indicator of hospital performance. As we explained in the paper, the mortality rate is too low to include in the DEA. The surgical volume is the only output factor for which we have collected data.
3) The fitness and efficiency of the DEA model was considered when we prepared the manuscript. Due to the manuscript length requirement, it was not possible to provide all details. The comment set of weights (CSW) method was used to calculate the optimal input weights by setting the bounds of variables and improving nonlinearity. More details were shown in the Supporting Information.
