# Supplementary material for: Surgical capacity, productivity and efficiency at the district level in Sub-Saharan Africa: A three-country study
Source: PLoS One. 2022 Nov 30;17(11):e0278212. doi: 10.1371/journal.pone.0278212 (PMC9710758; doi:10.1371/journal.pone.0278212)
Supplement: Attachment — Submitted filename: Response to Reviewers 2.docx [file pone.0278212.s009.docx]

**Comments from Reviewers**

**Reviewer 1**

*The authors have incorporated some comments from the first round of review, but I am not convinced by the responses to the comments # 1 and # 4.*

Thank you for your comments. Those two comments should be related to target units for inefficient DMUs and resources congestion.

Responding to the first point, we would like to reiterate that this study aimed to compare the difference between hospital performance measures by examining the determinant of hospital efficiency using data envelopment analysis (DEA). To ensure consistency in the analyses, the production factors used in the PIPES capacity index were used in the DEA.

We found that one reason the PIPES index and hospital efficiency presented almost opposite results was different weights assigned to the production factors. As what is shown in Table 5, the PIPES capacity index assigns higher weights to procedures, equipment and supplies that are less likely to be improved in a short time. DEA method weights infrastructure and personnel higher.

As to the ‘target units for inefficient DMUs’, we think what the reviewer meant was the efficient hospital with DEA score equal to one. Table 4 compared the difference in each production factor between high-efficiency hospitals (DEA score=1) and low-efficiency hospitals (DEA score<1), which provided information about ‘the target unit for inefficient DMUs to learn’. However, surgical efficiency is different from production efficiency. According to the production theory, any input, such as capital, wage, and raw materials, can be divided by units and then change the amount of inputs to produce the final good most efficiently. Surgical capacity, in turn, is hard to change. In Table 4, low-efficiency hospitals had higher infrastructure, procedures, equipment, and supplies than efficient ones. In theory, these inputs should be reduced in production for higher efficiency. But in practice, it is almost impossible to reduce infrastructure, equipment or supplies, and advanced skills surgeons have learned. Therefore, the only one possible way is to increase the personnel, i.e. more surgeons, doctors, and NPCs, since low-efficiency hospitals have lower personnel than efficient ones. And training NPCs has been proven as a good way in the literature to improve the number of surgically trained cadres in settings such as our study. That is why the conclusion was that “… investment in the surgical workforce, in particular, plays a central role in improving district hospital surgical productivity and efficiency”.

In response to the point about congestion, similar assumptions apply. Those district hospitals are the frontline providers of surgical services in rural areas. And Figure 3 illustrated that the performance results measured by efficiency score and surgical volume were similar. If more personnel can become the one input increased in those low-efficiency hospitals, then the slacks of other types of production factors will not be a restriction of efficiency but help to improve the output – providing more surgical services to rural populations.

**Reviewer 2**

*The authors have considered all my points in the revised article, so I think the revised article can be accepted.*

Thank you so much for your comments.
