# Supplementary material for: Surgical capacity, productivity and efficiency at the district level in Sub-Saharan Africa: A three-country study
Source: PLoS One. 2022 Nov 30;17(11):e0278212. doi: 10.1371/journal.pone.0278212 (PMC9710758; doi:10.1371/journal.pone.0278212)
Supplement: Attachment — Submitted filename: PONE.docx [file pone.0278212.s010.docx]

**Manuscript Number:** PONE-D-22-07365R2
**Manuscript Title:** Surgical capacity, productivity and efficiency at the district level in Sub-Saharan Africa: a three-country study

Dear Editor,

The authors have successfully responded to all my comments in the revised article, so I think the revised paper can be accepted.

Best regards,
